# An Exploratory Qualitative Study of Computer Screening to Support Decision-Making about Use of Palliative Care Registers in Primary Care: GP Think Aloud and Patient and Carer Interviews

**DOI:** 10.1177/21501327211024402

**Published:** 2021-06-12

**Authors:** Gill Hubbard, Kirsten Broadfoot, Clare Carolan, Hugo C. van Woerden

**Affiliations:** 1 University of the Highlands and Islands, Inverness, UK

**Keywords:** palliative care, decision-making, computer screening

## Abstract

**Objectives:**

This study aimed to understand factors that influence general practitioner (GP) use of automated computer screening to identify patients for the palliative care register (PCR) and the experiences of palliative care and this emerging technology from patients’ and carers’ perspectives.

**Methods:**

A computer screening program electronically searches primary care records in routine clinical practice to identify patients with advanced illness who are not already on a PCR. Five GPs were asked to “think aloud” about adding patients identified by computer screening to the PCR. Key informant interviews with 6 patients on the PCR and 4 carers about their experiences of palliative care while on the PCR and their views of this technology. Data were analyzed thematically.

**Results and Conclusions:**

Using computer screening, 29% additional patients were added by GPs to the PCR. GP decision-making for the PCR was informed by clinical factors such as: if being treated with curative intent; having stable or unstable disease; end-stage disease, frailty; the likelihood of dying within the next 12 months; and psychosocial factors such as, age, personality, patient preference and social support. Six (60%) patients/carers did not know that they/their relative was on the PCR. From a patient/carer perspective, having a non-curative illness was not in and of itself sufficient reason for being on the PCR; other factors such as, unstable disease and avoiding pain and suffering were equally if not more, important. Patients and carers considered that computer screening should support but not replace, GP decision-making about the PCR. Computer screening merits ongoing development as a tool to aid clinical decision-making around entry to a PCR, but should not be used as a sole criterion. Care need, irrespective of diagnosis, disease trajectory or prognosis, should determine care.

## Introduction

A review of deaths in high income countries suggests that between 69% and 82% of patients who die would benefit from palliative care.^1^ Early identification for palliative care is beneficial to patients.^2,3^ Early identification improves health-related quality of life; a Cochrane review of 7 randomized and cluster-randomized controlled trials of early palliative care for adults with advanced cancer reported that compared with usual/standard care alone, early palliative care significantly improved health-related quality of life at a small effect size (standardized mean difference (SMD) 0.27, 95% confidence interval (CI) 0.15 to 0.38) and lower symptom intensity (SMD −0.23, 95% CI −0.35 to −0.10).^2^ Early identification also reduces time in hospital; a retrospective cohort study comparing 230 921 decedents of early versus late palliative care found that fewer early recipients used palliative-acute care (42% vs 65%) with less days (mean days: 9.6 vs 12.0).^3^

There is no consensus internationally on defining or identifying people who need palliative care.^4^

A UK study found that General Practitioners (GPs) rely on a mixture of intuition, clinical knowledge and subjective judgment when deciding which patients to include on the PCR.^5^ Other factors influencing decision-making were a perception by GPs that patients were unlikely to benefit from being placed on the PCR and a reluctance to place patients who may be on the PCR for several years due to the unpredictability of their condition.^5^ However, these studies about GP decision-making used retrospective methods which are prone to recall bias. There currently exists little understanding of GPs’ cognitive processes as they occur in real-time, which means that we have only partial understanding of factors influencing the decision-making process for the identification of patients for the PCR.

Scotland uses palliative care registers (PCRs) in primary care to facilitate the assessment and review of patients with palliative care needs.^6,7^ The palliative care registers were introduced to try to improve the care and experiences of people at the end of their life. The PCR was expected to improve early identification of patients for palliative care and would include all patients with a life limiting condition, identified as having palliative care and end of life care needs, not just those with cancer. The PCR was also expected to drive quality of care because patients added the PCR would have been assessed and an initial care plan compiled and an electronic palliative care summary completed that would be made available to all professionals involved in the patient’s care in the out of hour’s period. In Scotland, in 2012 to 2014, 72% of patients who died of cancer and 32.5% of patients who died as a result of a non-malignant condition were listed on the PCR before death, respectively.^8^ This suggests that many people in Scotland, particularly those with non-malignant diagnoses, were not being placed on the PCR. Why patients are not being added to the PCR is unclear. Moreover, whether patients on PCR are identified earlier and have better quality of life and care experiences at the end of their life compared to those patients who have not been added to the PCR is uncertain. That is, we currently do not know the extent to which care is different for patients on the register.

In Scotland, a Supportive and Palliative Care Indicators (SPICT™) tool has been developed to facilitate identification of people at risk of dying in the next 12 months.^9^ SPICT™ includes 2 dimensions of illness to facilitate identification: clinical indicators of advanced conditions for 6 diseases (cancer, dementia/facility, kidney disease, respiratory disease, heart/vascular disease, and liver disease) and 6 general indicators of deteriorating health such as, 2 or more unplanned hospital admissions in the past 6 months. The tool therefore could potentially be used by GPs to identify patients for the PCR and in doing so, improve early identification for palliative care. AnticiPal is a computer algorithm which uses READ codes informed by SPICT™ to electronically search primary care records in routine clinical practice to identify patients with advanced illness who are not already on a PCR. The views of patients and carers (other countries use the term caregivers to define people who provide care for people in a voluntary capacity) about palliative care have been gathered^10^ but patients’ and carers’ views about use of automated computer screening for palliative care in primary care has not been extensively researched. A mixed methods study about computer screening for palliative care in primary care interviewed patients and carers about palliative care but did not report their views about the use of the screening tool to identify and manage patients for palliative care.^11^ Another mixed methods study about an automated mortality prediction tool for use in hospital reported that patients and carers perceived an advantage to their clinicians receiving an mortality prediction alert but voiced concerns over whether the tool would limit patients’ agency to make care decisions.^12^

The aim of this study was to understand patient identification for inclusion in PCRs, by eliciting real-time GP decision-making for identifying patients for the PCR. A further aim was to explore the experiences of palliative care and this emerging technology from patients’ and carers’ perspectives.

## Method

### Design and Setting

Think-aloud interviews were used in this exploratory qualitative study to understand contemporaneous GP decision-making about adding patients for the PCR while in-depth patient and carer interviews were undertaken to explore their views about automated computer screening tools and their experiences of palliative care while on the PCR. Think aloud methods are used because researchers cannot directly observe what someone is thinking and the method captures the verbalization of cognitive processes as they naturally occur in real-time. The method relies on the short-term memory in comparison to the use of interviews that enable an investigation of retrospective accounts of cognitive processes.^13^ A limitation of such retrospective verbalization of cognitive processes is participant reliance on long-term memory. Key informant interviews are qualitative in-depth interviews of people who have unique insights of a phenomena by virtue of first-hand experience.^14^ In this study, therefore, key informants were patients on the PCR and their carers.

The study was conducted in 4 General Practices in the north of Scotland.

### Recruitment and Eligibility

#### GPs

The sampling technique was purposive; 5 GPs from 4 different general practices located in both urban and rural practice involved in local palliative care improvement were identified and approached. Written consent by the GP was obtained before data collection, following a discussion of the study aims and provision of a Participant Information Sheet by a researcher. Locum GPs were excluded because they may lack familiarity with registered patients.

#### Patients and carers

Any patient aged 16 and over who was on the PCR and who a participating GP believed was competent to participate in an interview was eligible for the study. Carers of those patients were also eligible. Patients with a cognitive impairment and unable to give written informed consent were excluded from the study, although their carer was eligible. We aimed to recruit approximately 10 patients and 5 carers. This number of participants was considered realistic given the time and resources for the study whilst providing insights about patient and carer experiences and views about the PCR. The General Practice sent a letter and Participant Information Sheet to a patient on the PCR and/or their carer, inviting them to participate in the study. A GP may also have contacted a potential participant by telephone to discuss the study. GPs selected potential participants who met the eligibility criteria and who they perceived would be interested in the study. Hence, there was some potential selection bias, although the main focus was on a purposeful sample, linked to real life experience of being on a PCR. Only eligible participants who returned by post a signed consent form to the General Practice were included in the study.

### Ethical and Research Management Issues

The study was given ethical approval by NHS East of Scotland Research Ethics Service (REC reference 20/ES/0003) on 20th January 2020. NHS Highland management approval for non-commercial research was given on 31st July 2020. All study documentation, including data, were stored on University of the Highlands and Islands (UHI) computer system. Only researchers employed by the UHI had access.

### Data Collection Methods

The method of “thinking aloud” was used to explore decision-making by GPs about PCRs.^13,15,16^ We aimed to explore GP decision-making occurring in real-time since this is how decisions about the PCR are made in primary care. The think aloud was carried out by 2 researchers who both had a social science background and who had no relationship with the participating General Practices. A researcher arranged a time convenient to GPs to conduct a Think aloud via a video conferencing platform (a face-to face-approach was avoided to minimize risk of Covid-19 infection). Prior to the think aloud, the GP ran the AnticiPal program to generate a list of patients. The GP then verbalized every thought process that they became aware of while reviewing each patient on the list on the computer screen with the researcher present (online). The researcher aimed to get the GP to talk about what they were thinking, reading and searching as they worked on the task of deciding whether or not to include the patient identified by AnticiPal on the PCR. The researcher prompted the GP to verbalize their decision-making process using phrases “keep talking” or “Um-hummm” but otherwise stayed silent in order to avoid disrupting the thought process.^16^ This non-intrusive approach aims to only collect data dependent on GP use of their short-term memory and was video recorded. The “think aloud” interviews were conducted in September and October 2020 with the length varying between 48 and 60 minutes.

In contrast, key informant in-depth interviews were used to explore patient and carer experiences and perceptions of palliative care and use of computer screening to identify people for the PCR. Questions covered 3 main topics: (i) own experiences of palliative care and what this type of care means to them personally, (ii) views and experiences of being on the PCR, including what kind of person should be added to the PCR, (iii) views about a computer software package automatically using patient electronic records to identify patients who should be on the palliative care register. Interviews using a video-conferencing platform (a face-to face-approach was avoided to minimize risk of Covid-19 infection) were conducted between October 2020 and January 2021 with a median interview length of 33 minutes (including introductions), providing sufficient time for discussions without being too burdensome. All interviews were audio recorded with the participant’s consent. Patients and carers were interviewed separately. A previous study found that about half of patient and carer dyads preferred to be interviewed separately and that individual and joint or separate interviews both have strengths and limitations.^17^

### Data Processing and Analysis

The number of patients that GPs decided to add to the PCR were summed. The approach to qualitative data analysis was based on the researchers’ understanding that data are “interpreted” and open to interpretation rather than the truth “emerging” from the data and being “discovered”.^18^ Think Aloud data were analyzed using thematic analysis, which is a method for identifying, analyzing and reporting patterns within data.^19^ Given that there is very little evidence about decision-making by GPs about PCRs that currently exists, no robust theoretical framework to inform the analysis was used. Instead, a process of induction was used to allow for themes to emerge direct from the data without trying to fit it into a pre-existing coding frame or theoretical framework.^19^ The 2 researchers watched each think aloud video recording and coded the data together. These codes denoted what appeared of interest to the researchers and what words kept re-occurring throughout the dataset. All codes were grouped under key themes about GP decision-making relating to the PCR. To ensure consistency and trustworthiness, all data collection and analysis were conducted by 2 researchers.

To enhance the trustworthiness of the analysis and the researchers’ interpretation of the data, a focus group with the GPs who participated in the think aloud interviews was conducted for GP input and discussion of emerging themes. This focus group began with the researchers presenting the codes and key themes followed by a discussion so that the GPs could voice their interpretation of factors influencing the decision-making process.

Patient and carer interviews were transcribed and transcribed by a professional transcribing service. One transcript was coded by one of the researchers using in-vivo coding to construct a code sheet and then discussed with the other researcher. This code sheet formed the basis of transcript coding across the interviews. Separate code sheets were constructed for the patient and carer participants but significant thematic overlap was present. A process of induction was used to allow for themes to emerge direct from the data.^19^ As a technique to enhance trustworthiness of data, all coding was completed by 1 researcher with 2 other researchers reviewing transcripts and participating in data discussions to facilitate final interpretation of these data.

## Results

### Participants

Five GPs were recruited to the study and had been practicing for a median of 11 years; 2 were women. Three GPs did a think aloud individually and 2 GPs did the Think Aloud together. In total, 51 patients were discussed during the think aloud interviews (GP001 n = 8; GP002 n = 10; GP003/04 n = 21; GP005 n = 12).

Six patients and 4 carers from 4 different GP practices were interviewed. The average age of patient participants was 73.8 years. All patient participants interviewed for this study were already on the PCR (an inclusion criteria). All carer participants had a relative who was on the PCR. There were 2 patient-carer dyads in the sample, but otherwise participants responded individually. No age data was gathered for carers. For the purposes of presenting the results of this study, unique identifiers (GP, P (patient), C (carer)) were used consisting of a letter and numerical with quotations chosen by the authors of this manuscript to support each theme.

### GP—Think Aloud

Table 1 shows the proportion of patients identified by AnticiPal who GPs decided during think aloud should added to the PCR or not be added to the PCR (“No”). During the think aloud, GPs also referred to pre-palliative lists and so the number of patients that they decided to add to a pre-palliative care list is also reported. The table shows GP variation in level of agreement with AnticiPal for example, GP001 agreed with AnticiPal in 13% of cases compared with GP003/04 who agreed in 57% of cases.

**Table 1.** GP decisions about patients identified by AnticiPal.

Added to the Palliative Care Register?	GP practice 1		GP practice 2		GP practices 3 and 4 combined			GP practice 5		Total	
**No**	4	50%	7	70%	9	43%		8	67%	28	55%
**Yes**	1	13%	2	20%	12	57%		0	0%	15	29%
**Pre-palliative** **register**	3	38%	1	10%	0	0%		4	33%	8	16%
**Yes or Pre**	4		3		12			4		23	45%
**Total Patients**	8	100%	10	100%	21	100%		12	100%	51	100%

A range of factors informed GP decision-making about adding a patient to the PCR which broadly fell into 2 main thematic categories: (i) Clinical factors, and (ii) psychosocial factors. GP decision-making was multifactorial with social and individual dimensions of the patient’s context and wellbeing mediating clinical dimensions of the patient’s disease trajectory. Clinical factors for inclusion on the PCR foregrounded the decision-making process such as, whether the patient was being treated with curative intent, having stable or unstable disease, end-stage disease, frailty, and the likelihood of dying within the next 12 months. Psychosocial factors were also taken into consideration such as, a patient’s age, personality, patient preference about their care and social support.

The PCR was used as a tool for managing patients for palliative care. Patients could be added and taken off the PCR as and when for instance, a patient’s non-curative disease stabilized and de-stabilized:

*“This patient was on our palliative care list but we have since taken her off because she has reached a period of stability”* (GP003; P08).

Having stable disease did not preclude some patients being added to the PCR when other clinical factors were taken into consideration. One patient who did not have end-stage disease was frail and therefore the GP decided to add her to the PCR:

*“She’s doesn’t have anything what you would call end stage but she definitely has frailty. . . and so we should consider putting her on the PCR”*

(GP002:P08).

Another patient who had stable disease was considered eligible for the PCR because he had end stage disease:

*“He’s stable. . . it’s all about keeping him stable and treating his symptoms but obviously we’re not going to be able to reverse his disease. . . He should be on register because he has end stage liver disease but is young and because he’s young and doesn’t have any other comorbidities he is keeping going remarkably well with his terrible bloods. . . he probably should be on our PCR”*

(GP001; P02).

Being young was not sufficient reason for excluding this patient from the PCR but neither was being old an automatic justification for including a patient on the PCR. GPs weighed up a range of clinical and psychosocial factors during decision-making and therefore a patient’s age informed decision-making in the context of other factors. However, a GP, when reviewing a patient in their 90s who was currently fit, decided to add the patient to the PCR because being old meant that they were likely to die within the next 12 months:

*“So, she is one that if you are thinking about ‘could she die in the next 12 months?’ and she is an 94 year old, so in some ways yes, I suspect she might be someone who’s currently physically active, but at any point she could obviously have something. So in my point of view, probably worthwhile to have her on the palliative care register in terms of the way I would work it, but perhaps not the consensus amongst all the GPs at this practice.”*

(GP005; P12).

Other influencing factors in decision-making were patient personality and preferences for care. One GP decided not to add an elderly patient with non-curative disease on the PCR because the patient possessed stoicism:

*“She’s fairly elderly but pretty stoical”*

(GP001; P06).

Patient preferences for care and how they perceived themselves were also taken into consideration. A GP decided that a patient should not be on the PCR because the patient did not perceive that they were a palliative patient:

*“He’s probably not aware that we’d be thinking of him in a palliative care sense”* (GP001; P01).

Similarly, another GP discussing another patient said that she would not include her on the PCR because it did not match how the patient was managing her condition, which was to ignore her diagnosis:

*“She is on our palliative list because she has a metastatic tumour but actually she has quite stable disease. . . she is a lady who does not want to be reminded of her diagnosis and has very little contact with the surgery. . . she’s not had any end of life discussions with anybody but I think we are all very aware that if she deteriorate these discussions ought to be had but at the moment she is just living life and trying to ignore her diagnosis”* (GP003; P18).

Yet, if GPs believed that a patient’s condition warranted palliative care then despite a patient being fiercely independent or not perceiving themselves as needing palliative care, the GP was likely to decide that the patient should be included on the PCR. In other words, clinical factors when of significant concern appeared to trump patient preference:

*“Over all the years that I have known the lady a very difficult lady to manage. She is now 88 years old and leads an almost hermit-like existence in an isolated rural setting. . . she’s had previous quite significant medical issues. . . ischemic heart disease. . .a brief spell at A&E. . . a breast malignancy which she has refused treatment. . . I think to have her on the list would be totally appropriate”*

(GP004; P11).

GPs also referred to supportive relationships around patients such as spouses, relatives or professional carers, and the roles they played in their wellbeing during decision-making. Contact by spouses seemed to highlight to GPs that a patient’s condition was deteriorating and care needs were consequently increasing and therefore that the patient should be added to the PCR:

*“The striking thing with this patient is the significant increase in concerned calls from his wife and care team and community nurses that we’ve had during this year”* (GP004; P04).

Another GP decided that a patient who lived in sheltered housing should be put on the Practice’s pre-palliative care register rather than the PCR. What appeared to influence this decision was that his disease had stabilized and that he had good professional support:

*“He’s got really good support in the community; he’s in sheltered housing and has very good support from a support worker and they keep a close eye on him and they are happy to liaise with us if they have any concerns about him”*

(GP001; P07).

Hence, having good professional care and support appeared to influence the GP’s decision to keep him off the PCR.

### Patient and Carer—Qualitative Interviews

Of the 6 patients that were interviewed, 4 were unaware that they were on a PCR. Two out of the 4 carers were also unaware that their relative was on the PCR. Most participants did not know what type of care—palliative or end of life—they/their relative was receiving. Although all of the patients had non-curative disease and were already on the PCR, none of them self-identified as a PCR patient. The reasons why they did not believe they fitted the PCR patient profile are presented below and provide further insights about what participants believe is the purpose of the PCR and who therefore is eligible for the register.

One patient who had advanced cancer did not believe that she needed to be on the register because she currently felt well, although she did not rule out needing palliative care to manage pain at some point in the future:

*“Up till now I’ve really been fairly well. So maybe there hasn’t been the same need. . . I’m not actually suffering at this particular minute, pain from my cancer. I might not be seen as being somebody that was as much in need of palliative care than somebody who is actively in considerable pain from their cancer. . . I’m maybe not as actively needing medical help right now as I may come to be at some time in the future” (P002).*

The concepts of suffering and need featured in this participant’s reasoning for not necessitating being on the PCR. According to this participant, having non-curative disease was not automatic inclusion for the register; she suggests that the PCR is for persons in need of palliative care and in her opinion, she was not in need. The patient also understood that the future, although uncertain, was likely to lead to an increase in care. There are several ways that may help manage uncertain futures. One carer described how it was helpful for her to know where in the illness trajectory her husband was. She had been given a palliative performance scale checklist so that she could monitor his trajectory:

*“I’m the sort of person that I need to know what’s happening, I’m not one of those that don’t want to know and [the nurse in the care home] gave me a sheet, an assessment of the patient of a palliative patient. . . and it’s all the stages of palliative care, from the 100% level when you are normal and conscious level full, right down to 0%, which obviously is death. . . And it’s just something that’s very, very helpful for me” (C009).*

Another way of managing uncertainty is to predict life expectancy. A carer believed that palliative care should start when it was anticipated that the person had less than a year to live:

“*I think of palliative care being more towards the end of life, I would think if it’s within a year, they should be on it. But maybe palliative care starts earlier than that, I don’t know, but to me, if someone has only got a year to live, that should be a year of making things as easy as possible, even if it’s just coordinating the support they need” (C001).*

Despite having non-curative disease, feeling well and healthy and not needing higher levels of care seemed to be key reasons for exclusion from the register. One patient, despite not being able to breathe, did not believe she should be in the PCR because on the whole, she could manage to look after herself:

*“I feel there’s people more needier than me and maybe I will need it in the future. Oh, I don’t know. I really don’t know how to answer that because, as I say, I feel that other people might need it more than I do. Because apart from I can’t breathe, I think I’m pretty healthy. . . I feel that there’s people more ill than I am in need of it [the PCR] more. . . and I don’t need a lot of help, I need help to shower and they put cream on my legs at night but I can get up and get dressed, do my own meals, mind you most of them are microwave meals but I look after myself pretty alright” (P003).*

Although this participant needed carers for certain tasks, she did not believe that she fitted the category of patient who should be on the PCR. Hence, feeling well and being able to manage daily living in the context of living with non-curative disease was a conundrum for participants. The labels palliative care and end-of-life ran counter to lived experience. One carer explained why she found it difficult perceiving her relative as someone who was on the PCR because her relative was so well. This participant believed that palliative care was for people who were very ill and bedridden but her recent experience has taught her that this is not necessarily the case. Indeed, she now understood that people who are currently “well” are on the PCR:

*“Because my aunt is so well that I find it quite hard to take that on board, to be honest. If you asked me to define palliative care, I would say palliative care would be someone who was basically probably bedridden and very ill but now that I’m living through this, I can see that that’s not the case” (C001).*

Clearly, participants perceived that the PCR was for people who were very ill and no longer able to manage by themselves as opposed to people like themselves who seemed to be doing okay (from their perspective) in spite of their non-curative condition. Participants took a person’s ability to manage into consideration when deliberating on what kind of patient should be included on the PCR. One participant said that the PCR is especially important for those who live alone and need looking after:

*“People who have a diagnosis for the end-of-life, I presume. Like I’ve been told my cancer is terminal, it canna be fixed, it’s not a case of living with it, in case it gets worse, but that’s the sort of people who will have to be on it, somebody who has got to be looked after. Especially anyone on their own, I’d hate to deal on my own with any kind of problem like this. So they should certainly be on the register” (P003).*

Other participants said it was important that a person was added to the PCR if they could not manage to look after themselves:

*“Well basically anyone that can’t do for themselves, really. I mean once you become incapable” (P004).*

Thus, the concept managing was also important when defining who should be on the PCR.

### Attitude to Automatically Generated PCR Computer Records

Participants were asked their views about whether a computer algorithm should decide which patients should be added to the PCR. Participants identified health professionals as key to decision-making about the PCR, not a computer algorithm. One participant believed that the most important decision-makers were the multi-disciplinary team in charge of their care:

*“I think it should be a discussion between patient and – patient, consultant and GP” (P010).*

Some participants added family members to the list of people who should be involved in decision-making about the PCR:

*“But I think the doctors and nurses would be very important – and the carers I think, too” (P005).*

One patient dismissed outright the use of computer algorithms to assist in decision-making:

*“Well I would think it would be your doctor and your family. . . .I’ve no faith in computers at all” (P004).*

However, other participants could see that computer screening to identify patients for palliative care may have a role to play in decision-making. One patient re-iterated the importance of the GP in decision-making about the PCR, although she was not averse to a computer assisting the GP’s decision-making:

*“I personally wouldn’t have any problems with that [computer algorithms to identify patients for the PCR] because I’ve no real problems about, it would be done, I assume, under all the usual protocols, data protection etc. I don’t suppose I would have any problem with that. I’m not sure that I would like the input of the GP to be lost completely because that’s your first line of contact normally and they know you really better than anyone else, really. I can see my doctors at any time within reason whereas the consultants, I’m only seeing – or speaking to – on a three-monthly basis” (P002).*

A carer could see that a major advantage of the use of computers—although not necessarily its use to identify patients for the PCR—was it could facilitate decision-making by recording key decisions. She could visualize this being particularly useful given that clinicians responsible and managing a patient’s care can change:

[on the use of computer software] *“I think that’s a good idea, yeah. Because doctors change and surgeries - people move on and nurses move on and so if that information is sort of, you know, there, then people can refer to it and that’s a good thing because they can refer to that and say, “Okay, this is what they thought then” and, you know, if anything changes, they could consult the family or the carer or whoever but certainly, you know, that’s what’s been complied at that point and, you know, it’s probably important to have that there, uh-huh” (C005).*

## Discussion

### Summary and Comparison with Existing Literature

This is the first study to capture GP decision-making about the PCR in real-time. The study has also identified that, in our sample, 60% of patients/carers were unaware of the PCR and if they/their relative was on it. Moreover, patients and carers in the sample did not know or did not perceive as important, what type of care—palliative or end of life—they were receiving or the label being used to define their care. This is similar to a previous study which found patients and carers have limited understanding of the concept palliative care.^11^ Nonetheless, all patients and carers in the study were fully aware that they or their relative had non-curative disease and that treatment was no longer with curative intent. More important than the use of the label palliative care was how making sure that a person was not suffering or in pain and that their care needs were being met. The study highlights that decision-makers for the PCR and patients and carers face a conundrum—participants on the PCR interviewed in this study perceived that they were not currently in need of palliative care and therefore not suitable for the PCR because they felt relatively well and healthy and could manage with no or minimal care.

GPs weighed up a multitude of clinical factors—cognizance of long-term conditions, stability of disease, stage in the disease trajectory (eg, end stage), frailty, likelihood of dying within the next 12 months, and treatment (eg, active treatment)—in conjunction with an assessment of the patient’s character, care preferences and social support during decision-making about the PCR. A diagnosis of a progressive or life-limiting illness did not imply automatic placement on to the PCR; instead, GPs searched for evidence of a deterioration in the disease trajectory or expected that the patient would die within the next 12 months to justify placing a patient on the PCR. Similarly, patients and carers perceived that care need—not diagnosis per se—was key to deciding who was eligible for the PCR.

GPs not only used their medical expertise but their knowledge about the patient, including the patient’s social circumstances and their preferences such as, if the patient wishes to consider themselves as someone who requires palliative care. Previous studies have reported that some GPs were reluctant to label patients as “palliative” due to the association of the term with death and dying and subsequent loss of hope.^5,20^ It is likely that there will always be a subjective element in decision-making regarding the PCR which incorporates personal knowledge of the patient, their social context and perceived patient preferences.^5^

In this study, computer screening (AnticiPal) resulted in GPs deciding to add an additional 29% of patients to the PCR. This suggests that such technology is useful and merits further development. In conjunction with the PCR, General Practices used other lists to manage patients such as, pre-palliative lists. The notion of a pre-palliative list for patients who would need palliative care at some point in the future was also found in a previous study.^11^ However, there seems to be significant inter-individual variation between GPs in their threshold for adding a patient to a PCR or a pre-palliative list. This variation is not surprising; a systematic review has highlighted the lack of consensus for defining palliative care patients and ambiguity in the use of the adjective palliative.^21^ The use of pre-palliative registers, although useful in principle, may increase this variation in practice. In contrast, the potential advantage of a computer generated list of patients for the PCR at least for comparative purposes, is the extent to which a standardized threshold can be applied across different General Practices. Yet, there were differences of opinion among patients and carers about the role of computer screening in decision-making about who to add to the PCR. Even those who did not dismiss the use of computer screening still wanted the GP to have the final say in decision-making about care as the person who knows each individual patient and their personal contexts of care best.

### Implications for Practice

Illness trajectories and prognostication have been described for people with progressive chronic illnesses but none of these trajectories are able to pinpoint the right time from a patient and carer perspective to start palliative care.^22,23^ A recent systematic review reported that screening tools differed significantly in their ability to identify patients with potential palliative care needs with sensitivity ranging from 3% to 94% and specificity ranging from 26% to 99%.^24^ Our study suggests that, from the patients’ and carers’ perspective, the right moment is not a question of improving the accuracy of trajectory or prognostication but monitoring and responding to care need to ensure that there is no pain and suffering. Thus, tools that improve the accuracy of predicting pain and care need may prove more useful in the long-term.

This study suggests that GP and members of the clinical the team should remain central to decision-making about who is eligible for the PCR; computer screening while a useful tool to prompt identification of those with possible palliative care needs is not a substitute. Indeed, the developers of AnticiPal and SPICT™ suggest that these tools should be used to help identify people whose health is deteriorating and should act as a trigger for clinicians to assess a patient for unmet supportive and palliative care needs and to plan care.^25^ Such tools are therefore not designed for a patient’s automatic inclusion on the PCR in the absence of expert review as indicated by several participants in this study.

Finally, experiencing wellness while having non-curative disease was described by patients and carers and requires a sensitivity on the part of clinicians to the evolution of this experience and the necessity of ongoing conversations as awareness and acceptance that anyone’s experience was likely to change over time. This concept of wellness when tied with independence and mobility challenged definitions of who should be considered palliative and therefore on the PCR, and also made deciding the most appropriate timing of palliative care conversations challenging. This means that carers and patients should engage in conversations with their primary care clinicians of care received in the here and now as well as anticipating care for the future. It should be noted that care in the here and now was often conducted by the GP and carers in a state of vigilance as they monitored any clinical signs of a turning point in care that would trigger something else planned or needed. Such an approach requires continuity of care, with the GP, and in their absence, multi-disciplinary team members familiar with the patient and their context. This continuity is associated with reduced mortality, but appears to be at odds with the policy direction in the UK.^26-28^

### Study Strengths and Limitations

As far as we know, this is the first study where a think-aloud approach was used to capture real-time decision-making by the GP for patient identification for the PCR. It is also novel in its exploration of patient and carer perspectives on the use of automated computer algorithms to generate a list of patients for palliative care. It is important to acknowledge that this exploratory study involved only a small number of GPs, patients and carers in one Scottish health board and as such, generalizations should be made with caution. Further, coding was not done independently for the think aloud data and patients and carers were not asked to comment on the researchers’ interpretation of the interviews.

### Conclusion

Assessing care need, rather than diagnosis, disease trajectory, or prognosis *per se*, is most important to patients with non-curative disease in primary care. Computer screening focusing on clinical indicators may assist in decision-making for palliative care alongside GPs subjective judgments about patients’ psychosocial factors. Decision-making about care should take account of patient and carer preferences about their care and the label (if any) that they wish to give for the type of care that they receive.
